# The Role of Selected Serpins in Gastrointestinal (GI) Malignancies

**DOI:** 10.3390/jcm11206225

**Published:** 2022-10-21

**Authors:** Sara Pączek, Barbara Mroczko

**Affiliations:** 1 Department of Biochemical Diagnostics, University Hospital in Bialystok, Waszyngtona 15A St., 15-269 Bialystok, Poland; 2 Department of Biochemical Diagnostics, Medical University in Bialystok, Waszyngtona 15A St., 15-269 Bialystok, Poland; 3 Department of Neurodegeneration Diagnostics, Medical University in Bialystok, Waszyngtona 15A St., 15-269 Bialystok, Poland

**Keywords:** serpins, cancer, gastrointestinal malignancies, biomarkers, serine protease inhibitors

## Abstract

Gastrointestinal (GI) cancers, which are a diverse group of malignant diseases, represent a major healthcare problem around the world. Due to the lack of specific symptoms in the early stages as well as insufficient diagnostic possibilities, these malignancies occupy the leading position in the causes of death worldwide. The currently available tests have too many limitations to be part of routine diagnostics. Therefore, new potential biomarkers that could be used as diagnostic and prognostic factors for these cancers are still being sought. Among the proteins that might fit this role are serpins, which are serine protease inhibitors. Although the serpins themselves have been known for many years, they have recently become the centre of attention for many authors, especially due to the fact that a number of proteins in this family are involved in many stages of neoplasia formation, from angiogenesis through tumour growth to progression. Therefore, the aim of this review is to present the current knowledge about the significance of serpins in GI malignancies, especially their involvement in the development and progression of oesophageal, gastric, pancreatic and colorectal cancers. This review summarises and confirms the important roles of selected serpins in the pathogenesis of various GI cancers and also points to their promising roles as therapeutic targets. However, due to the relatively nonspecific nature of serpins, future research should be carried out to elucidate the mechanisms involved in tumour pathogenesis in more detail.

## 1. Introduction

### 1.1. Gastrointestinal (GI) Cancers

The digestive tract consists of many interconnected sections responsible for maintaining the human body in full health [1]. Gastrointestinal (GI) cancers are a diverse group of diseases that include oesophageal cancer (EC), gastric cancer (GC), pancreatic cancer (PC), liver cancer (LC), gallbladder cancer (GBC) and colorectal cancer (CRC) [2]. According to the data presented by the American Cancer Society, in 2018, there were almost 2 million new cases of GI cancers, accounting for over 30% of total cancer deaths, according to estimates [3]. These tumours have been observed to occur in both sexes and represent the most common types of cancer among men and the second most common in women. In most cases, tumours of the GI tract are neoplasms with relatively poor prognoses, which is reflected in the small differences between the numbers of cases and deaths in particular years of follow-up [4]. Although the incidence of some types of GI cancer has decreased, this group of malignancies remains a major public health challenge [5]. The incidence of GI cancers also presents geographical variation. The CRC incidence is higher in Western Europe and North America, while the LC and GC incidences are higher in Asia and Africa. However, PC is more common in the populations of industrialised countries. Thus, in Europe, it is much more frequent in the Nordic countries than in the Mediterranean ones [6,7,8]. The evaluation of the risk factors for GI cancers has shown that more than half of all these cancers are caused by modifiable risk factors, such as high-percentage alcohol consumption and tobacco smoking, whose roles in carcinogenesis have been confirmed for many types of histological malignancies [9,10,11]. Many studies have shown that prevention in the form of regular physical activity, an appropriate diet rich in fibre and protection against viral infections, as well as drug elimination, reduce the risk of GI cancers [11,12].

The clinical symptoms of GI malignancies vary depending on the location as well as the tumour stage and mostly affect fatigue, which is followed by malaise, pain, sleep disturbances, a lack of appetite, dyspnoea and nausea [13,14,15,16]. However, the later a diagnosis is made, the lower the chance of a cure.

The diagnostic process for patients with GI cancers is based on endoscopic evaluation. However, the auxiliary examinations cover the scope of imaging diagnostics as an important tool in both diagnosis and staging. In the diagnostic process for patients with GI neoplasms, apart from imaging methods, it is also recommended to perform laboratory tests. In addition to visualizing the patient’s general condition, they allow the assessment of the classical tumour markers of GI neoplasms [17,18,19]. These include such markers as carcinoembryonic antigen (CEA), tumour antigen 19-9 (CA 19.9), tumour antigen 72.4 (CA 72.4) and tumour antigen 50 (CA 50). Unfortunately, these markers are not useful in the early detection of these tumours due to their poor diagnostic criteria such as low diagnostic sensitivity and specificity [20,21,22,23].

The treatment options for GI cancers primarily depend on the type and stage of the cancer and the patient’s age, general health and preferences. The most common treatments for GI today include chemotherapy with targeted therapy, surgery and radiation therapy. A relatively new concept is neoadjuvant therapy, which can consist of chemotherapy, radiation therapy or hormone therapy [24,25,26,27]. One example of the use of neoadjuvant therapy techniques is rectal cancer. In this case of locally advanced neoplasm, the CXRT (chemo-radiation therapy) is used, which allows usually for the reduction of local recurrence, but usually does not affect distant metastases. This treatment is followed by surgery for 8–12 weeks [28].

The prognosis of patients with GI neoplasms remains unchanged. Although current treatment strategies have developed, patients’ quality of life has only slightly improved. The limited efficacy of therapy in patients with advanced disease reflects an incomplete understanding of the molecular basis of GI carcinogenesis. Currently, the most important prognostic factors for patient survival are the histological degree of the tumour and the tumour stage at diagnosis, including the depth of tumour infiltration and the involvement of regional lymph nodes. In addition to these clinico-pathological parameters, GI cancer biomarkers are being intensively researched and validated. However, the low sensitivity and specificity of the available biomarkers limit their usefulness, especially in early stage screening or in distinguishing between aggressive and benign tumours. For this reason, research is ongoing to find new biomarkers that would allow for early cancer detection and assessment of response to therapy [29,30,31]. 

### 1.2. The Structure and Functions of Serpins

**Figure 1:**
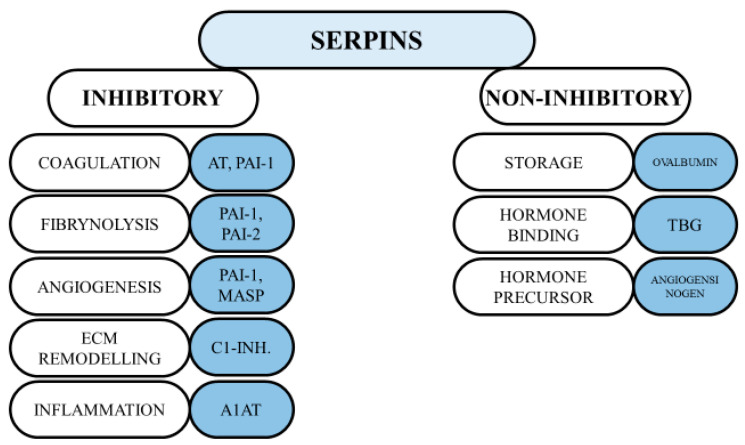
The inhibitory and non-inhibitory function of serpins [43]. A1AT: alpha-1-antitripsin; AT: antithrombin; PAI-1: plasminogen activator inhibitor-1; PAI-2: plasminogen activator inhibitor-2; MASP: maspin; TBG: thyroxine binding globulin.

The activity of proteases appears to play a key role in the survival of multicellular organisms. These enzymes are responsible for breaking peptide bonds, which in turn leads to irreversible post-translational modifications of proteins [32]. An example of such proteolytic cascades is inflammation and coagulation, which, if inappropriately activated, can lead to clotting disorders and inflammation in the host. Thus, it is not surprising that protease activity is regulated by various inhibitors, including the serine protease inhibitors [33]. Serpins (SERine Proteinase INhibitors) are the largest superfamily of protease inhibitors, representing about 2–10% of circulating plasma proteins [34]. They generally consist of approximately 350–400 amino acid residues, with molecular masses of 40–100 kDa. The core of a serpin’s structure is described as highly conserved, which has been found to be important for its functions [35]. Native serpins present two common features: five-stranded beta-sheets A are located in the middle of the molecule, and a flexible RCL (reactive centre loop) is located on the top of the molecule. The currently used nomenclature for serpins consists of two parts: alphabetic and numerical designations. However, this nomenclature does not refer to any of their evolutionary relations, but rather to the order of their discovery [36]. The comprehensive phylogenetic analysis of the eukaryotic serpin family divided them into 16 clades—from A to P. However, many serpins bear alternative names that were given before the current classification was proposed [37]. Serpins are mainly found as extracellular molecules, although some of them, such as clade B serpins, may act inside cells due to their absence of N-terminal secretory signals [38]. Among the newly characterised functions of these proteins, the regulation of cell proliferation appears to play a major role [39]. Accordingly, several serpins have been found to be overexpressed in tumour cells [40]. Most serpins are responsible for the inhibition of serine proteases, but some of them have additional functions that inhibit some representatives of cysteine proteins, such as caspases as well as cathepsins [41]. Serpins have been also called the “workhorses” of the human body because they play extremely important roles in the regulation of diverse biological activities. Moreover, these proteins are involved in several physiological processes such as blood coagulation, inflammation and immunity [42]. Therefore, serpins have been also classified according to their functions. The inhibitory and non-inhibitory functions of serpins are presented in Figure 1 [43]. Apart from their physiological implications, serpins have also been found to be involved in the pathogenesis of many diseases. Diseases caused by both mutations and dysfunction of the serpins themselves have been termed serpinopathies [44]. Most serpin-related diseases are the result of serpin polymerisation and aggregation, but there are several other types of disease that are caused by their mutations. The most common inherited disease is alpha-1-antitrypsin deficiency [45]. Recent scientific work has defined two potential pathways by which serpins are responsible for tissue damage. The first one concerns the accumulation of polymers, which leads to endoplasmic reticulum stress and inflammation (e.g., liver cirrhosis) [45]. The second mechanism involves the loss of serpin function, which results in uncontrolled activity of proteases. An example is the hyperactivity of elastase resulting from alpha-1-antitrypsin (A1AT) deficiency, leading to emphysema [46].

## 2. Materials and Methods

A comprehensive literature search was performed by using the MEDLINE/PubMed database covering the period up to March 2022. The authors used following search strategy: studies were limited to those in the English language, and duplicates and all non-significant manuscripts (i.e., papers that did not concern the selected serpins/cancers and biomarkers) were excluded. Initially, the authors used the keyword “serpins” (68,332 studies). Then, the following keywords were employed: “serpins AND cancer” (8657 studies) and “serpins AND gastrointestinal cancer or GI cancer” (744 studies). Due to the small number of records, the review focused on selected cancers, which most often appeared in the results, taking into account their abbreviations: “serpins AND oesophageal cancer or EC or ESCC” (15,664 studies), “serpins AND gastric cancer or GC” (291 studies), “serpins AND pancreatic cancer or PC” (4530 studies) and “serpins AND colorectal cancer or CRC” (390 studies). The analysis of the obtained results identified the five most common serpins, which are described in this review. Bearing in mind the different names of the serpins, the aliases as well as abbreviations were also included in the key words, e.g., “serpinA1 or A1AT or alpha-1-antitripsin AND CRC or colorectal cancer”. A literature search on the remaining serpins (vaspin, maspin, PAI-1 and PEDF) and tumours (GC, PC and CRC) was conducted following the same pattern.

## 3. Results

### 3.1. Serpins in Cancer

Oncogenesis is the process by which normal cells become cancerous. It is characterised by both genetic and cellular changes, as well as abnormal cell division [47]. Under normal conditions, the balance between cells’ proliferation and their apoptosis preserves the integrity of both tissues and organs [48].

Serpins can bind to and interact with numerous molecules, and their function has therefore evolved from simple serine protease inhibitors to biological molecules with complex properties. These proteins are associated with the progression or remission of various cancers, making them valuable in diagnostic and therapeutic applications [49]. However, the role of serpins in cancer, as well as their mechanisms, is subject to much controversy [50]. In the present review, the involvement of these molecules in tumours will be described based on two examples: plasminogen activator inhibitor-1 (PAI-1) and pigment epithelium-derived factor (PEDF).

**Figure 2:**
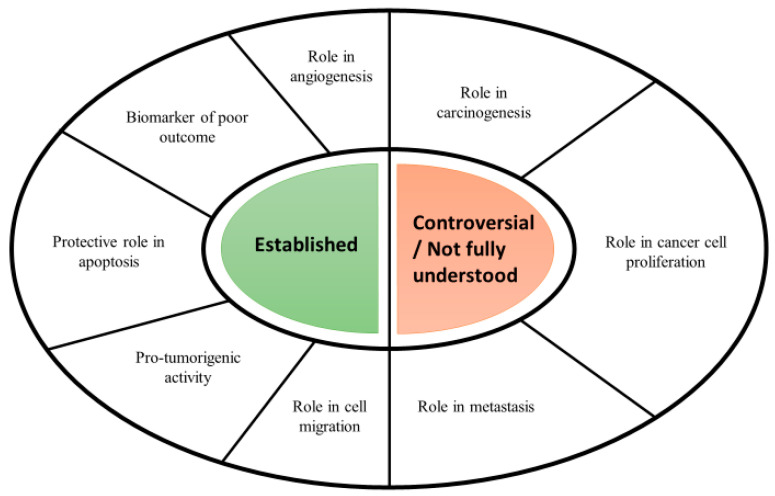
The role of PAI-1 in cancer [56].

PAI-1 appears to be the most studied serpin in cancer biology. It has been suggested that it plays a key role in cancer, mainly through its association with urokinase-type plasminogen activator (uPA) and the extracellular matrix (ECM) protein vitronectin [51]. However, the involvement of PAI-1 in human cancer is multifaceted and, through a number of different mechanisms, may affect the maintenance of proliferative signals, angiogenesis, invasion and the formation of distant metastases. A relatively recent discovery is the participation of PAI-1 in cancer-related inflammation [52]. Experimental studies conducted in both animal and human models have shown that inflammatory cytokines such as interleukin-6 (IL-6) or tumour necrosis factor alpha (TNF-alpha) increase the expression of PAI-1 [53]. This suggests that the increase in PAI-1 is a consequence of an inflammatory state. The latest research supports this theory, leading to strong conclusions that this serpin is both a consequence and a cause of inflammation, especially by increasing interleukin-8 (IL-8) production [54]. However, many reports have shown that elevated levels of PAI-1 are observed in inflammatory conditions not associated with cancer, such as sepsis or arthritis [55]. The role of PAI-1 in cancer is presented in Figure 2.

PEDF is a member of the serpin superfamily, which can act directly on tumours by inducing differentiation to a less malignant phenotype as well as promoting the apoptotic death of tumour cells [57]. Moreover, this protein can also inhibit the proliferation of neoplastic cells, and has antimetastatic effects by inhibiting the invasion and migration of neoplastic cells. In this regard, it is worth bearing in mind that many reports show that reduced PEDF levels are associated with tumour progression, and the exogenous administration of PEDF in animal models results in tumour growth inhibition and prolonged survival [58]. The mechanisms underlying the regulation of the behaviour of cancer cells by PEDF are mainly based on the interaction of this serpin with various receptors on the cell surface, which triggers a number of signalling pathways [59].

### 3.2. The Role of Serpins in GI Cancers

Much evidence proves that several serpins play roles in various cancers [40,60]. Among all the serpins, A1AT, waspin, maspin, PAI-1 and PEDF have been shown to make the greatest contributions in cancer [60,61,62,63]. Therefore, they are described more fully in the present manuscript. The involvement of selected serpins in GI cancers is presented in Table 1. 

#### 3.2.1. SERPIN A1 (Serpin Family A Member 1)—A1AT (Alpha-1-Antitrypsin)

A1AT is a plasma glycoprotein in the alpha 1 fraction of globulins, and is considered to be one of the most potent serine protease inhibitors [85]. About 70–80% of this protein is produced and secreted into the bloodstream by liver cells, while the rest is synthesised by monocytes and macrophages in the respiratory system [86]. A1AT is one of the acute phase proteins; hence, its serum concentration fluctuates significantly and may increase rapidly in the course of inflammatory responses [87].

A number of studies have demonstrated that A1AT might be a potential biomarker in several cancers including GI cancers [61,88].

The study performed by Bernacka K et al. evaluated the concentrations of A1AT in the sera of GC patients in comparison to healthy volunteers [89]. The authors observed an increased concentration of A1AT in the blood of patients at the initial stage of the disease, and a correlation between the concentration of this protein and the stage of the disease, which suggests that it may have utility in the early detection of this cancer [89]. Similar results were demonstrated by Yang J et al., who revealed the upregulation of A1AT in the serum samples of GC patients [64]. In addition, the level of A1AT was found to be significantly higher in the gastric juice of GC patients in comparison to healthy controls [64]. The opposite results were obtained by Wu JY et al., who found decreased concentrations of A1AT in GC patients when compared to the control group [65]. Due to the fact that A1AT has an anti-inflammatory effect in vitro, thus contributing to the inhibition of the synthesis of pro-inflammatory cytokines such as IL-8 or TNF-alpha, it is suggested that the decreased level of this serpin may be responsible for the lack of the suppressive effect of these cytokines [65].

The association between the serum levels of A1AT and survival in PC patients was studied by Trichopoulos D et al. The authors indicated that the serum levels of A1AT represented a statistically significant prognostic indicator of PC [66]. Moreover, it was shown that the variable values of the difference in serum A1AT concentration at the time of diagnosis influenced the implications for the difference in survival [66]. 

The relationship between A1AT and CRC still seems controversial, as many studies show both higher and lower blood levels in CRC patients compared to control groups [67,68,69]. One study that evaluated the significance of A1AT in CRC patients was performed by Jaberie H et al. The authors found that the plasma levels of A1AT were significantly higher in CRC patients compared to those in a control group consisting of healthy volunteers, and those concentrations were found to positively correlate with the tumour stage [67]. These results are in line with previous studies involving the proteomic analysis of serum samples, which showed that A1AT outperformed serum CEA in distinguishing CRC patients from healthy controls [68]. However, it is worth remembering that elevated levels of A1AT in the blood are also observed with both benign neoplasms and other malignant neoplasms.

#### 3.2.2. SERPIN A12 (Serpin Family A Member 12)—Vaspin

Vaspin (visceral adipose tissue-derived serine protease inhibitor—VASP) is a serpin that belongs to the family of adipokines. It is encoded by the *OL-64* gene, and the locus is on chromosome 14 (14q32.1). VASP is a protein with a molecular weight of 45.2 kDa and about 40% homology with A1AT [90]. Increased concentrations of VASP have been identified in obesity, and it was also shown that elevated levels are associated with diabetes mellitus, metabolic syndrome, obesity and coronary artery diseases [91]. Interactions between VASP, cell apoptosis and proliferation have been already indicated. These relations, on the one hand, are key processes for homeostasis and, on the other hand, through their dysfunction and dysregulation, may predispose to the development and progression of many diseases, including cancer [92,93]. However, the exact link between VASP and cancer is still not entirely clear. According to current knowledge, it is known that most adipokines are capable of promoting tumour progression mainly by enhancing the proliferation and migration of tumour cells as well as inflammatory pathways [94,95]. Another mechanism is the participation of these proteins in anti-apoptotic pathways, which may consequently lead to the formation of cancer metastases [93,96]. With regard to GI cancers, the role of VASP remains a mystery. Thus far, scientists have studied its significance in relation to hepatocellular carcinoma (HCC) and CRC.

The study by Pazgan-Simon M et al. investigated the relationship between adipokines and metabolic abnormalities as well as the severity of liver dysfunction. The authors assessed the VASP serum levels in HCC patients in comparison to a control group as well as the association between those concentrations and HCC grade and progression [70]. The authors showed a positive association between increased VASP concentrations and HCC, with upregulated VASP concentrations in the sera of patients with LC when compared to healthy volunteers [70].

Fazeli MS et al. assessed the relation of serum VASP concentrations in patients with CRC [71]. Significantly higher levels of VASP were observed in the research group compared to the healthy controls. This finding suggests that this serpin may also be involved in carcinogenicity [71]. Studies report that VASP, by regulating the PI3K/Akt signalling pathway, has the potential to protect vascular endothelial cells from free fatty acid-induced apoptosis [71,72]. Therefore, it is suggested that VASP may prevent apoptosis, by influencing the above pathway [71,72].

#### 3.2.3. SERPIN B5 (Serpin Family B Member 5)—Maspin

Maspin (mammary serine protease inhibitor—MASP) is a 42 kDa serine protease that belongs to the family of ovalbumin-like serpins called ov-serpins [97]. MASP has been proven to be expressed in the epithelia of many human organs such as the thymus, breast, small intestine and colon [98]. Although this serpin is commonly expressed in the cytoplasm, it can also be detected in the nucleus [99]. It has been proven that this protein plays an extremely important role in both the promotion of tumour cells’ adhesion and the inhibition of their mobility [100]. Although MASP has been characterised as a tumour suppressor, its overexpression is suggested to play a key role in tumour progression as well as enhanced aggressiveness for various cancers such as breast, gastric and pancreatic [101,102]. However, some investigations have reached opposite conclusions for CRC [103]. In the context of GI tumours, this serpin has been studied in patients with ESCC, GC, PC and CRC [104,105,106].

The association between MASP expression and better overall survival in ESCC was studied by Wang et al. The authors revealed that cancer patients who showed low or moderate MASP expression had lower postoperative survival rates than those who showed high MASP expression [73]. This leads to the conclusion that the transient regulation of MASP in the early stage of ESCC development may be a kind of defence mechanism, which prevents further progression to more malignant phenotypes, ultimately inhibiting tumour progression.

Wang MC et al. evaluated the significance of MASP expression in GC progression [74]. The expression of MASP was significantly negatively associated with both the invasive depth and metastasis, but not with tumour size and TNM stage [74]. Decreased MASP expression has been suggested to contribute to GC progression through mechanisms such as reducing cell apoptosis as well as facilitating angiogenesis [74]. Therefore, MASP has the potential to be considered as a marker of the biological behaviour of GC.

The study by Snoeren N et al. aimed to identify genes and proteins in CRC patients with liver metastases that may correlate with early disease recurrence [75]. Based on immunohistochemical analysis, elevated MASP expression was observed in the central tumour cores and also associated with poor histological grade in CRC patients. In addition, MASP was shown to be an independent predictor of the time to recurrence and survival specifically for CRC in stage III of the disease [75].

#### 3.2.4. SERPIN E1 (Serpin Family E Member 1)—PAI-1 (Plasminogen Activator Inhibitor-1)

PAI-1 is a glycoprotein member of the serpin superfamily encoded by SERPINE1 that plays an important role in the plasminogen/plasmin system [107]. It is produced by hepatocytes, endothelial cells, megakaryocytes and platelets [108]. It has been shown that PAI-1 circulates in the blood in three different forms: active, latent inactive, or in the form of complexes with uPA, tPA, or vitronectin [109]. Elevated PAI-1 levels have been shown to be a risk factor for diseases such as thrombosis and atherosclerosis [110]. This molecule plays an extremely important role, among others, in cardiovascular diseases and, as a member of the serpin family, in cell migration [111]. However, there is evidence that, apart from playing important roles in cell adhesion, migration, or invasion, this protein is capable of inducing tumour vascularisation [56]. It has been shown that PAI-1 is highly expressed in many types of neoplastic tissues and consequently contributes to tumour progression by promoting cell proliferation and neoplastic metastasis [112].

The association between invasiveness, migration and prognosis as well as PAI-1 expression in ESCC patients was evaluated by Zhang Y et al. [76]. The authors revealed statistically significant differences in PAI-1 expression between ESCC tissue and normal oesophageal mucosa [76]. Additionally, the expression of the tested protein in patients with lymph node metastases (LNMs) was statistically significantly lower than that in patients without them [76]. The above suggests that PAI-1 may be a new molecular marker for predicting both the progression and prognosis of ESCC. The results show that extensive research on the PAI-1 protein could lead to its application as a therapeutic target [76]. The expression of PAI-1 in patients with ESCC was also studied by Wang D et al. [77]. The authors showed that the overexpression of PAI-1, through interaction with LRP1, could increase both the invasion and migration of ESCC cells, and also promote ESCC progression [77]. 

Chen H et al. evaluated the expression of the PAI-1 protein in the tissue as well as levels of PAI-1 in the plasma of CRC patients [78]. The protein expression was significantly increased in CRC tissue taken from patients with liver metastases when compared to samples from patients without liver metastases [78]. The authors also showed that the plasma PAI-1 levels were higher in CRC patients with liver metastases in comparison to the control group [78]. Furthermore, those results correlated with the tumour size, liver metastasis and LNM. 

The study by Märkl B et al. investigated the association between the tissue level of PAI-1 and aggressive behaviour of colon cancer [79]. It was shown that increased PAI-1 was significantly associated with the occurrence of distant metastases [79]. In addition, multivariate analysis revealed PAI-1 as an independent predictive factor of distant metastases [79].

#### 3.2.5. SERPIN F1 (Serpin Family F Member 1)—PEDF (Pigment Epithelium-Derived Factor)

PEDF is 50-kDa serpin, secreted primarily as a soluble glycoprotein. It has been found to have a broad spectrum of biological roles in many pathologies [113]. It is known as an important antitumorigenic, antiangiogenic and antimetastatic factor in several cancers [114,115]. A number of studies have demonstrated the potent tumour-suppressive effects of PEDF [116,117]. The molecular mechanisms of PEDF’s protection against cancer progression remain of interest to scientists [118]. Some theories suggest that it is related to the inhibition of tumour neovascularisation but also to the fact that PEDF is capable of downregulating vascular endothelial growth factor (VEGF) [119]. However, many studies still show many contradictions regarding the pro- and antitumour significance of this serpin. Previous research suggests that PEDF is involved in the induction of apoptosis through several pathways, suggesting it has potential for use in the treatment of cancer [120].

The significance of PEDF in GC was assessed. The study performed by Zhang Y et al. found that, in the case of GC, tumour growth and angiogenesis were inhibited by the injection of PEDF via the downregulation of both hypoxia-inducible factor 1 alpha (HIF-1 α) and vascular endothelial growth factor (VEGF) [80]. However, this serpin could not directly induce tumour cell apoptosis. The opposite results were obtained by Aksoy EK et al., who demonstrated statistically significantly higher PEDF concentrations in the sera of GI patients compared to a group comprising patients with precancerous lesions (PCLs) [81].

Principe DR et al. showed decreased PEDF expression in human pancreatic tissue samples when compared to a control group comprising healthy volunteers [82]. In human PC, decreased levels of PEDF have been observed, both in the tissue and in the serum. This decrease is potentially related to increased tumour angiogenesis, as well as the occurrence of liver metastases and a poorer prognosis [82].

The expression of PEDF in CRC patients was investigated by Wågsäter D et al. [83]. The level of the PEDF protein was assessed in both the plasma and tumour tissue, as well as healthy tissue, obtained from CRC patients [83]. It was shown that the concentration of PEDF in the plasma in the group of patients with CRC was significantly lower compared to that in the control group. Studies by Ji D et al. have shown that decreased PEDF expression in both sera and CRC tissues correlated with liver metastases [84]. In addition, cohort studies showed that patients with lower levels of PEDF in their CRC tissues had worse overall survival (OS) than patients with high levels of these proteins [84]. This led to the conclusion that PEDF could serve as a prognostic marker as well as a potential therapeutic target in CRC.

### 3.3. Serpins as Target for Cancer Therapy and Future Perspectives

Serpins are proteins with a proven role in many processes related to carcinogenesis, such as the proliferation of cancer cells and tumour progression. Moreover, their significance in the remission of selected neoplasms has also been demonstrated, which makes them a potential target for use in monitoring and, above all, in cancer therapy. An example of a serpin that can be used in this way is PAI-1. Although this protein has been shown to both reduce and increase tumour growth, recent studies suggest that blocking PAI-1—by modulating the function of the urokinase activator receptor—reduces cancer cell migration and survival [55,121].

Another anti-cancer serpin is MASP, which increases the sensitivity of cancer cells to apoptotic stimuli. According to PAI-1, MASP inhibits the migration of neoplastic cells. However, while significant experimental data support the role of MASP as a potential tumour suppressor, clinical data on the prognostic implications of its expression have led to conflicting results. This highlights the great need for a better understanding of MASP’s relationships in cancer biology. Moreover, research into recombinant MASP has led to the consideration of the question of whether rMaspin itself may be a viable candidate in the fight against cancer [100,122,123]. In contrast to the intracellular pro-apoptotic activity of MASP, extracellularly, its activity in vitro appears to be cytostatic, as cells have been found to regain both migratory activity and mobility after the discontinuation of rMaspin treatment. Some research assessed the prognostic value of MASP expression following fluarocyl treatment in CRC. These studies showed that low nuclear MASP expression appeared to be an independent predictor of benefits from adjuvant chemotherapy in patients with CRC [124].

PEDF is a multifunctional molecule that is highly antiangiogenic and pro-apoptotic. It also has the ability to inhibit tumour growth and distant metastasis by inducing tumour apoptosis and inhibiting tumour angiogenesis [60]. Recent studies have clearly shown that, in addition to the obvious antitumor activity of exogenous PEDF, changes in endogenous expression are associated with the progression of many malignancies [125]. Immunohistochemical analysis of PEDF expression in human neoplasms revealed that an increase in PEDF expression correlates with a better prognosis, which supports the idea that the determination of PEDF might be a prognostic factor in the treatment of cancer. Therefore, determining the level of PEDF in a tumour or the serum at the time of diagnosis could be an excellent source of information for delineating more or less aggressive treatment protocols to improve the therapeutic outcomes in cancer patients [60,120,126].

In designing serpin therapy, it is critical to consider issues such as protein half-lives, goals and clinical aspects that influence the development of metastatic proteins. Despite promising results, further research into the efficacy and mechanisms of serpin-mediated antitumor activity is needed and warranted in order to develop new alternatives for the treatment of cancer.

## 4. Conclusions

GI cancers are among the most common cancers in the world. The morbidity and mortality rates are extremely high for both women and men. Therefore, there is an urgent need to conduct further research on early GI tumour biomarkers. One group of proteins that seems to be controversial in terms of showing both pro- and anti-cancer properties is the serpins. This paper summarises the significance of selected serpins in the pathogenesis of GI neoplasms, confirming that selected serpin proteins play extremely important roles in the development and prognosis of GI cancers. The present manuscript demonstrates that, of all the serpins, A1AT may serve as a prognostic biomarker of PC and is observed to be increased in the initial stages of GC. In addition, elevated concentrations of vaspin are observed in the serum of HCC and CRC patients, suggesting that it plays a role in the pathogenesis of those cancers. Moreover, many of the described serpins such as maspin and PEDF are interesting targets for anti-cancer therapies, since lower concentrations and expression of them are associated with worse overall survival and the presence of distant metastases. This review summarises the role of serpins as biomarkers with potential use in GI cancers. Moreover, it has been shown that these proteins in GI cancers are useful not only in the context of screening or diagnosis, but also in prediction and monitoring of those malignancies. In addition, the assessment of diagnostic and predictive factors will most likely change the current stage of GI cancers. However, due to the non-specific nature of serpins and the additional observation of elevated levels of them in non-cancerous diseases, further research should be carried out to determine the most appropriate use of these molecules.

## Figures and Tables

**Table 1:** The roles of selected serpins in GI cancers.

Serpin	Alternative Name	GI Cancer	Significance	References
SERPINA1	A1AT	GC	❖ Increased blood concentration in initial stages of cancer ❖ Serum upregulation in cancer in comparison to control group ❖ Decreased serum concentrations in cancer compared to control group	[64,65,66,67,68,69]
		PC	❖ Statistically significant prognostic indicator of cancer	
		CRC	❖ Significantly increased plasma levels in cancer patients compared to control group ❖ Correlation between its plasma concentration and tumour stage	
SERPINA12	Vaspin	HCC	❖ Increased concentrations in the sera of cancer patients	[70,71,72]
		CRC	❖ Significantly higher concentrations in the sera of cancer patients	
SERPINB5	Maspin	ESCC	❖ Low expression associated with lower survival rate	[73,74,75]
		GC	❖ Expression significantly negatively associated with metastasis	
		CRC	❖ Independent predictor of recurrence	
SERPINE1	PAI-1	ESCC	❖ Significant difference in expression between cancer and normal tissue	[76,77,78,79]
		CRC	❖ Significantly increased protein expression in CRC metastatic tissue when compared to non-metastatic tissue ❖ Increased serum concentration associated with distant metastasis	
SERPINF1	PEDF	GC	❖ Significantly higher serum concentrations in cancer patients compared to control group	[80,81,82,83,84]
		PC	❖ Decreased expression in cancer tissue when compared to healthy volunteers	
		CRC	❖ Decreased expression correlated with liver metastasis ❖ Significantly lower plasma concentrations in cancer patients when compared to control group	
